# Cognitive behavioral treatment for disordered gaming and problem gambling in adolescents: a pilot feasibility study

**DOI:** 10.48101/ujms.v127.8693

**Published:** 2022-08-08

**Authors:** Frida André, Isak Einarsson, Elisabeth Dahlström, Katalin Niklasson, Anders Håkansson, Emma Claesdotter-Knutsson

**Affiliations:** a Faculty of Medicine, Department of Clinical Sciences, Lund, Lund University, Lund, Sweden; b Outpatient Department, Child and Adolescent Psychiatry Clinic, Region Skåne, Sweden; c Psychiatry, Faculty of Medicine, Department of Clinical Sciences, Lund, Lund University, Lund, Sweden; d Region Skåne, Malmö Addiction Centre, Gambling Disorder Unit, Malmö, Sweden; e Region Skåne, Child and Adolescent Psychiatry, Regional Outpatient Care, Lund University Hospital, Lund, Sweden

**Keywords:** Gaming, gambling, CBT, relapse prevention

## Abstract

**Background:**

Disordered gaming and problem gambling (DG/PG) are associated with a range of functional impairments as well as psychiatric comorbidity. With the proliferation of digital gaming apps aimed at children and adolescents, which involve in-game purchases, there is increasing evidence that DG/PG are on the rise in this age range. The behavior can be detected in youth presenting at school-based health clinics and community psychiatric clinics. Cognitive behavioral therapy (CBT) is one of several recommended treatments for adults, but little evidence is available for the efficacy of this approach in adolescents with DG/PG.

**Aim:**

To evaluate the acceptability and feasibility of a CBT-based intervention developed for adolescents with DG/PG, which can be delivered in routine psychiatric care facilities.

**Methods:**

Adolescents who were patients at a child and adolescent psychiatry service were screened for DG/PG. Those aged 12–17 years with pronounced symptoms were invited to participate in a 7-week CBT program called Relapse Prevention. Nine adolescents agreed to participate and five consented to repeated assessments of outcome (pre-, post-treatment, and 6-month follow-up). In addition to acceptability and satisfaction with treatment, symptoms of DG were assessed with standardized interview and self-report measures.

**Results:**

There were no dropouts from the treatment. Participants who completed treatment and all outcome assessments reported satisfaction with the treatment. The participants showed fewer symptoms of DG after treatment, and the proportion who met criteria for computer game addiction decreased from 56 to 0%. There was no reduction in the number of participants who met criteria for PG.

**Conclusion:**

This study provides preliminary evidence for the acceptability and feasibility of a CBT-based intervention for DG/PG in adolescents. Preliminary data suggest that the treatment may be effective for DG but not PG. Further studies are needed to evaluate the efficacy of this approach for both conditions.

## Introduction

Research on the potentially harmful effects of gaming has grown in the last two decades (1, 2), with the field taking a big step forward with the introduction of Internet Gaming Disorder (IGD) as a tentative diagnosis in The Diagnostic and Statistical Manual of Mental Disorders, fifth edition (DSM-5) (3). Gambling for money is only allowed for adults by Swedish law (4). However, there is evidence that the behavior exists also among the younger part of the Swedish population. An epidemiological study from 2018, of Swedes aged 16 years and above, found that roughly 1% of those aged 16–17 years reported some degree of problem gambling (PG) (5). The prevalence of the diagnosis of IGD and its relationship to PG in Swedish youth have not yet been investigated (6). Comparable studies in neighboring countries report GD prevalence ranging from 0.6 to 5.5% (7), and a study from 2015 presented an overall European prevalence of 1.6% (8). Major international studies show the prevalence of disordered gaming (DG), a category broader than IGD as defined in ICD-11, ranging from 1.3 to 6.8% (2).

The availability of digital gaming applications (apps) aimed at children and adolescents has increased to a great extent during the past decades. It is increasingly common that these gaming apps encourage the player to purchase items, the so-called ‘loot boxes’, that give the player advantages in the game, blurring the line between gaming and gambling. A population survey of Swedes aged 15 years and above found an association between DG and PG (8). Two studies following a cohort of Swedish 13- and 15-year-olds over 3 years found an association between DG and PG among adolescents, but DG per se did not seem to predispose the youth to PG (9, 10). Whether PG is present or not, adolescents with DG often present as compulsive, with elevated levels of health and psychiatric complaints and with impaired academic functioning (6, 9). The presence of commonly occurring mental health conditions in youth, including attention deficit hyperactivity disorder (ADHD), depression, and anxiety, appears to be potential risk factors for DG (10). Among adolescents registered to child and adolescent psychiatry (CAP) clinics, those with ADHD and autistic spectrum disorder (ASD) are overrepresented among those seeking additional help for DG (10, 11). The authors speculate whether the repetitiveness and immediate reinforcement that characterize digital gaming may place these youth at increased risk for developing DG.

It is important to note that a wide range of scales are used for assessing DG in research and clinical settings, and this contributes to considerable variability in prevalence and comorbidity estimates (9, 10). Many studies use the criteria for pathological gambling to define pathological gaming (2, 10). One of the most frequently used questionnaires for assessing DG in adolescents is the Game Addiction Scale for Adolescents (GASA) (9, 12–14). The seven-item scale is based on the DSM-5 criteria for pathological gambling, with items corresponding to salience, tolerance, mood modification, withdrawal, relapse, conflict, and problems (14). The DSM-5 suggests that half or more of the criteria should be met when diagnosing pathological gamblers (3). However, DG gaming researchers point out that the tolerance, mood modification, and cognitive salience criteria correspond more to engagement and not necessarily addiction, while the contrary applies to the withdrawal, relapse, conflict, and problems criteria (12, 13, 15, 16). They suggest that a potential diagnosis of DG should distinguish engaged gamers from problem- and addicted gamers by accentuating the latter four criteria (withdrawal, relapse, conflict, and problems) (12, 13).

There is no gold standard treatment for either DG or PG in young people (6). As such, there are no national guidelines in Sweden for their screening or treatment, or on whether youth with DG/PG should be assisted by psychiatric or social services. Nevertheless, it is clear that some children and adolescents who engage in frequent digital gaming and gambling need professional help to gain better control over their behavior (6, 17). Cognitive behavioral therapy (CBT) is often identified as a first-line treatment for DG, but the available evidence is limited. A recent meta-analysis (18) identified 12 treatment trials of CBT for DG, the majority of which were carried out in Asia. Across trials, CBT was delivered in either group or individual formats and was focused on helping patients to recognize triggers (cue-induced cravings) and to develop beliefs and behaviors that increased their motivation to quit or reduce gaming (11, 19). There was considerable heterogeneity across studies, but large effect sizes were observed for DG and comorbid depression, and moderate effect sizes for comorbid anxiety. While relatively few of the participants in the trials were below 18 years of age, the authors found no evidence that treatment was less effective for adolescents than adults.

The present pilot study is a part of a larger research program aiming to develop knowledge on DG in youth and to design, implement, and evaluate a treatment for DG patients recruited from child and adolescent psychiatry (CAP) clinics across southern Sweden (Region Skåne).

First aim: to explore the feasibility of delivering relapse prevention (RP) as treatment of DG in a CAP setting.

Second aim: to explore the outcome of RP on DG.

Third aim: to explore the outcome of RP on PG.

Fourth aim: to explore how the participants experienced the treatment.

## Materials and methods

A treatment model of DG/gambling based on RP has been developed (20). RP is a CBT-based form of treatment, originally developed for the treatment of alcohol problems in adults. Currently, RP is also used for addiction problems in both adults and adolescents regarding alcohol, drugs, tobacco, and gambling (21). In this study, the RP model is further adapted to enable treatment of DG among children and adolescents. The number of sessions was reduced, and the treatment was provided individually instead of being group based, to better fit the CAP sample’s needs and preferences. The original idea was to provide RP as a group treatment as well, but none of the participants was interested in such an arrangement.

During the spring of 2020, patients within the CAP outpatient and inpatient care were screened for DG, originally to collect data for a study prior to the current one (22). Clinicians (psychologists and psychiatrists) were systematically provided with questionnaires to distribute to their patients. The survey reached 144 children and adolescents between 8 and 18 years of age. Seven individuals were excluded due to participating without sharing their social security number or not answering the items on gaming, leaving 137 individuals (22). Roughly, 30% (*n* = 29) met the criteria for DG, according to the tentative criteria suggested by the DSM-5 (3, 22). Those aged 12–17 years were requested to participate in an interventional study. Altogether, nine children and adolescents (13–17 years), eight (89%) male and one (11%) female, were included. Among the nine participants, seven (78%) met the criteria for DG at the start of the study. The participants were assessed with GASA regarding gaming (14) and Control, Lying, and Preoccupation (CLiP) regarding gambling (23), before treatment, after treatment, and at 6 months follow-up after treatment. The primary outcomes of interest were acceptability and feasibility of the treatment, and secondary outcomes were DG symptoms assessed via the GASA. A potential effect on PG, assessed via CLiP, constituted a tertiary outcome. Information on the participants’ gender, age, housing situation, and main diagnosis was also collected. An informed consent was obtained from the participants and their guardian/guardians.

### GASA

One of the most used measures for DG is GASA (9). The scale is based on seven of the nine DSM criteria for PG (salience, tolerance, mood modification, relapse, withdrawal, conflicts, and problems) (14). The DSM-5 suggests that at least half of the criteria should be met for a diagnosis of gambling addiction (3), hereafter mentioned as the DSM approach (DSMA). Many of the gaming scholars emphasize the importance of differentiating highly engaged but harmless gaming from a truly pathological gaming behavior (12, 13, 15, 16, 24–26). The core approach (CA) is a method that accentuates the criteria that includes negative consequences, with the aim of separating highly engaged gaming from pathological gaming. The core approach implies that the endorsement of each of the ‘core criteria’ of relapse, withdrawal, conflicts, and problems implicates addictive gaming, while endorsement of two or three core criteria implicates DG, and the endorsement of one or less core criteria but each of the peripheral criteria (salience, tolerance, and mood modification) implicates engaged gaming (12, 13).

### CLiP

In 1999, Gerstein et al. developed a screening instrument for gambling problems – the NORC Diagnostic Screen for Gambling Problems (NODS) (27). The 17-item questionnaire corresponds to the DSM-IV criteria for PG and yields a score ranging from 0 to 10. NODS-CLiP includes the NODS-items involving loss of control, lying, and preoccupation – the ‘CLiP’ (23, 28). The questionnaire has been shown to exhibit excellent sensitivity and specificity for NODS constructs (23, 28). Answering ‘yes’ on at least one item indicates PG (23, 28).

### Participant evaluation

After completing the treatment, all participants were offered a chance to evaluate the treatment anonymously. The response rate for the evaluation was 56%. The evaluation consisted of eight questions developed by the authors. The first question was ‘How much has the treatment helped you in regulating your gaming, 0–10?’. The respondents were supposed to mark a value between 0 and 10 in which 0 corresponded to ‘Not at all’, 5 corresponded to ‘Medium’, and 10 corresponded to ‘Extreme’. The second question was ‘How much did the gaming bother you before the treatment, 0–10?’ and the third question applied the same but regarding after the treatment. The fourth question concerned motivation to participate in the treatment, also answered by marking a value between 0 and 10. Question 5 was ‘Was it easy to understand what we talked about?’ to which the respondent could answer ‘No’, ‘Yes, a little’ or ‘Yes, a lot’. Question 6 contained three sub-questions with the heading ‘The treatment contained different parts, how much has the following helped you:’. The first part applied to the gaining of more knowledge about game addiction, the second part applied to the tasks that were done together with a therapist, and the third part was about the homework. The respondent answered these questions with ‘Not at all’, ‘Quite a bit’, ‘Partly’, ‘Quite a lot’, or ‘Very much’. Questions seven and eight were answered in free text and requested: ‘What was the best parts of the treatment?’ and ‘What could be improved before future treatments?’.

### The treatment

There is no consensus regarding the treatment method for PG. Together with four experienced psychologists in the field, we, therefore, developed a manual that we wanted to try out, primarily in the present pilot study and subsequently in a full RCT. We developed a manual based on previous knowledge in the field of addiction and in the field of child and adolescent psychiatric treatment. We used RP as a base and adapted the manual for children and adolescents. Clinicians (psychiatrists and psychologists) with training in CBT were educated in RP and were, throughout the treatment, supervised by experienced RP clinicians. The treatment was adapted to fit the participants’ primary problem behavior, either gaming or gambling. Patients who met the criteria for DG (according to tentative criteria from the DSM-5) were offered a chance to participate in an RP-based treatment intervention at their local clinic or, where applicable, at an adjacent clinic or online through video-link. The treatment model is manualized and includes a motivating and relapse-preventative approach, in which the therapist explores not only the patient’s exhibited and undesirable behavior but also their motivation for change, their goal, and which events, emotions, and thoughts induce the gaming behavior or result in continuation of the behavior or relapse (20). The treatment is individual and consists of seven sessions of 45 min over a period of 7 weeks.

### Analysis

Statistical analysis and calculations were performed in SPSS (IBM SPSS statistics version 27). To evaluate the treatment efficacy, the difference in GASA score among before treatment, after treatment, and at follow-up was analyzed with a one-way repeated measures ANOVA. McNemar’s test was used to evaluate if the proportion of participants who met the cut-offs for different levels of gaming changed after completed treatment. The gaming categories that were counted and compared were engaged gaming (CA), problem gaming (CA), addicted gaming (CA), and problem gaming (DSMA).

### Ethical considerations

The participants’ anonymity has been protected by de-identifying all participants’ contributions. Any risks of participating in the study are considered minor. The risk of being exposed to physical harm by participating in the study is considered to be extremely limited. The patient is not left alone either during or after the assessment. All participation was voluntary, and the patients were informed that they could cancel their participation at any time without giving a reason. The current study was approved by the Ethics Committee (Dnr: 2019-04797).

## Results

### Sample characteristics

Table 1 shows the sample characteristics. Eight participants (89%) were male, and the age range was 13–17 years, whereof four (44%) were 17 years old. ADHD was the most common diagnosis, followed by depression. Anxiety was the main diagnosis only for one participant. Equally many lived with cohabitant and separated parents, while one individual reported other conditions. Before treatment, seven individuals (78%) met the criteria for (at least) problem gaming, regardless of the use of the DSMA or the core approach. Before treatment, three individuals (33%) answered affirmative to questions about gambling.

**Table 1:** Sample characteristics, at the start of the study.

	*n*	%
**Gender**		
Male	8	89
Female	1	11
**Age**		
13	1	11
14	1	11
15	0	-
16	3	33
17	4	44
**Diagnosis**		
ADHD	5	56
Depression	3	33
Anxiety	1	11
**Housing situation**		
Cohabiting parents	4	44
Divorced parents	4	44
Other	1	11
Engaged gaming (CA)^1^	1	11
Problem gaming (CA)^1^	2	22
Addicted gaming (CA)^1^	5	56
<Engaged gaming (CA)^1^	1	11
**Problem gaming (DSM)^2^**		
Yes	7	78
No	2	22
**Problem gambling^3^**		
Yes	3	33
No	6	67

ADHD: attention deficit hyperactivity disorder; CA: core approach; DSM: .

1 According to the Game Addiction Scale (GAS) – CA, core approach.

2 According to the Game Addiction Scale (GAS) – DSM approach.

3 According to the CLiP.

### Outcome 1 – RP efficacy on gaming

**Figure 1:**
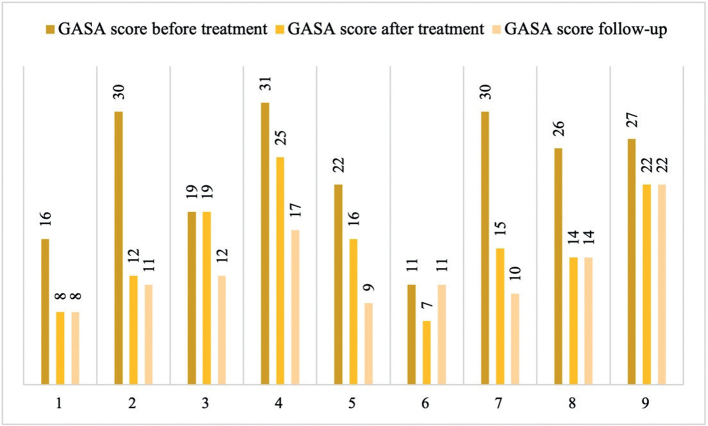
Individual GASA score before treatment, after treatment, and at follow-up.

Figure 1 illustrates how the GASA score changed among before treatment, after treatment, and at follow-up. Table 2 shows the results of the repeated measures ANOVA. The mean GASA score before treatment was 24, after treatment 15, and at the time of follow-up, it was 13. The repeated measure analysis shows that the mean GASA score differed significantly between before and after treatment, and so did the GASA score between before treatment and at follow-up. The mean score after treatment did not differ significantly from the GASA score at follow-up. Tables 3 and 4 show that the proportion of participants who showed very few DG symptoms that they did not even meet the criteria for engaged gaming, according to the core approach, was significantly higher at the time of follow-up. The proportion of participants who rated their gaming too low that they did not meet the criteria for problem gaming according to the DSMA also increased significantly.

**Table 2:** McNemar’s test for X^2^ comparisons of the prevalence of gaming categories between before treatment and follow-up.

	Before treatment % (*n*)	Follow-up % (*n*)	*p*
**Core approach**			
Engaged gaming	11 (1)	0.0 (0)	-
Problem gaming	22 (2)	11.1 (1)	1.000
Addicted gaming	56 (5)	0.0 (0)	-
Less than engaged gaming	11 (1)	88.9 (8)	0.016
**DSM approach**			
Problem gaming	78 (7)	11.1 (1)	
No-problem gaming	22 (2)	88.9 (8)	0.031

**Table 3:** Estimates of mean GASA score, before treatment, after treatment, and at follow-up.

Mean GASA score	Mean	95% confidence interval
Before treatment	23.6	18.2–29.0
After treatment	15.3	10.7–20.0
Follow-up	12.7	9.3–16.0

**Table 4:** One-way repeated measures ANOVA. Comparison of GASA-score among before treatment, after treatment, and at follow-up.

Mean GASA score		Mean difference	*p*	95% confidence interval for difference
Before treatment	After treatment	8.2	0.003	3.8 to 12.6
	Follow-up	10.9	0.001	5.9 to 15.9
After treatment	Before treatment	−8.2	0.003	−12.6 to −3.827
	Follow-up	2.7	0.092	−0.5 to 5.9
Follow-up	Before treatment	−10.9	0.001	−15.9 to −5.9
	After treatment	−2.7	0.092	−5.9 to 0.5

### Outcome 2 – RP efficacy on gambling

Among the nine participants, three individuals (33%) met the criteria for PG before treatment and just as many thereafter. Two individuals who gambled before the treatment did no longer gamble after completed treatment, while two individuals who did not gamble before treatment did endorse gambling after completed treatment. Only one individual affirmed gambling for money both before and after treatment.

### Outcome 3 – participants’ evaluation

The evaluation is illustrated in Figures 2–4. The participants who answered the evaluation reported that the treatment had helped them to regulate their gaming. Most of the participants stated that gaming disturbed them more before the treatment than after. However, one individual scored higher on item 3 (How much did the gaming bother you after the treatment?) than item 2 (How much did the gaming bother you before the treatment?). The motivation to participate in the treatment varied with scores ranging from 4 to 10 (4–6, 10). Most thought it was very easy to understand what the therapists were talking about. One individual did not find it easy to understand. Question 6 was about how much the different parts of the treatment had helped the participants, and the majority was positive to all the parts (increased knowledge about DG, tasks with a therapist, and homework). The participants stated in free text that ‘a lot had been fun’, ‘everything, altogether was good’, and ‘it helped, taught me a lot’. One participant stated that the most positive thing about the treatment was ‘the conversation, he had a different view on DG than me, it was good to talk about it’. Suggestions for improvement were formulated such as ‘More hands on, try to reduce gaming concretely, more game-free days earlier in the treatment, better access to the material, have all the material attached so you do not lose it (like a book)’ and ‘maybe more conversations, the opportunity to go deeper into certain areas instead of getting another task’.

## Discussion

The main purpose of this pilot study is to evaluate the acceptability and feasibility of a CBT-based treatment for DG in adolescents recruited from CAPs in southern Sweden. Within the framework of the current study, therapists have been trained in RP, and a small number of CAP patients have been admitted for treatment. In summary, the results of this study indicate that the treatment might be effective. Those who participated in the evaluation throughout reported that the treatment helped them to regulate their gaming, and the participants rated their gaming significantly lower after completing the treatment.

CBT-based treatment of DG is probably the most studied method, and the results are considered promising (18, 29, 30). However, the overall evidence is still described as insufficient for definitive conclusions, and further research is required (10, 29, 30). Furthermore, adults seem to respond better to treatment than youths, and the evidence to determine whether CBT treatment reduces time spent on gaming is still described as insufficient (18). Furthermore, there is an ongoing debate as to whether the absolute time spent on gaming is a relevant measure of outcome or whether it is the ability to control the gaming that matters (18). In line with that reasoning, several previous studies have emphasized the importance of avoiding pathologizing computer gaming per se, but only the gaming behavior that results in negative consequences. The core approach aims to separate extensive gaming from potentially pathological gaming by underlining the criteria that implicitly include negative consequences (12, 13). This study showed that the proportion of participants who met the criteria for computer game addiction, according to the core approach, decreased by 100% after treatment. Also, the proportion of participants who showed few symptoms of DG that they did not meet either the criteria for engaged gaming, according to the core approach, or the criteria for problem gaming, according to the DSMA, both increased significantly. The fact that so many different measurement approaches exist in previous research (2, 9) complicates conclusions regarding our results in comparison to others.

The main purpose of this study was to evaluate RP as a treatment for DG, serving as a precursor to a larger RCT about RP for DG. The secondary purpose of this study was to evaluate the effect on gambling. Two individuals who affirmed gambling before treatment denied gambling after treatment, while two other individuals answered affirmative to questions on gambling only after treatment. This outcome should be interpreted in the light of the fact that the treatment was adapted to fit the participants’ primary problem behavior, either gaming or gambling. Gambling is illegal for children in Sweden (4). Games with or without money elements are closely related phenomena in the sense that financial transactions, the so-called ‘loot boxes’, are common in computer games, and computer game-like virtual environments occur where games about money take place. Furthermore, a link between the consumption of ‘loot boxes’ and gambling for money has been demonstrated, and ‘loot boxes’ have been described as a gateway to gambling, among adults (31). The participants in this study did not specify what kind of gambling they endorsed, but nevertheless, they were too few in number for conclusions to be drawn regarding any positive effect of RP on the gambling intended. Gambling among children is still an unexplored phenomenon, and the high prevalence shown in this specific sample motivates extended exploration.

The participants who responded to the evaluation reported throughout that the treatment helped them to regulate their gaming. Yet, the participants did not consistently report that gaming disturbed them less after treatment than before. If a behavior disturbs more after a treatment than before, the treatment could possibly be considered a failure, even though the behavior has become easier to regulate. This discrepancy could be explained by an increased insight into negative aspects of one’s own gaming behavior because of the treatment, and the long-term effect could possibly be more undividedly positive. However, this is an aspect that requires further investigation. The participation was voluntary, and one could expect that everyone who committed to the treatment would have been at least moderately motivated. The fact that two individuals rated their motivation lower than five (corresponding to medium) raises questions as to whether the motivation was carried primarily by the child/adolescent participating or by their guardian. It would be of interest to investigate whether the level of motivation to participate in the treatment had an impact on the outcome.

The pilot study served as a precursor in designing an RCT. The design of an RCT is a joint work between academia and CAP in Region Skåne. Recourses, both human (clinicians) and also localities, are of great importance. The pilot study was performed during the first wave of the COVID-19 pandemic, and our following work with the RCT was heavily affected by the pandemic.

### Limitations

This study should be interpreted in the light of its limitations. It is a pilot version of the final RCT study that includes a control group, with the aim to modify and optimize the conditions for the final RCT study. An obvious limitation is the limited number of participants, which obstructs a deeper investigation of potentially underlying factors that affect the outcome of treatment. Furthermore, the feasibility approach of this pilot study and the fact that the relatively pronounced changes in this limited sample (such that the number of patients who fulfilled the addiction criteria for gaming using the core approach decreased by 100%) unfortunately mean that adequate power for a subsequent RCT is difficult to calculate. In addition, this study does not include a control group, and it is, therefore, possible that factors other than the treatment contributed to the suggested improvement in terms of DG symptoms, such as the attention suddenly received from the parent accompanying to the CAP clinic once a week. Also, since other treatment studies used different measurement scales, the results cannot be compared with others. The fact that only one of the study’s nine participants was female must also be mentioned as a limitation. DG has been described as a male problem (32), but women are engaging in gaming to an increasing extent, and more research is needed to evaluate not only gender differences in DG but also potential gender differences in treatment outcomes. Another limitation is the manualized structure of the treatment. One treatment will not fit all, and in the future, one has to take into consideration the diversity of the patients regarding both maturity and comorbidity. A patient with ASD might not benefit from the same treatment as a patient with depression regardless of their similarity in PG. Only five respondents (56%) chose to participate in the evaluation, and the generalizability of the results of the evaluation to the entire sample is questionable. The evaluation included two open-ended questions, resulting in three and four freely formulated responses, respectively. The low number of quotes complicates a more pronounced qualitative design, which, otherwise, would have been appealing and could probably also have served as an interesting contribution to the study’s content. In creating the RCT that will follow this pilot study, we need to address the motivational aspect since the participants will be randomized to RP treatment. In the RCT, we plan to add a qualitative part regarding both the participants’ evaluation and the clinicians. Altogether, the results of the evaluation may be regarded as an opportunity for insight into how the treatment can be experienced, and it contributes to valuable insights, to implement in the future study design.

## Conclusion

This study provides preliminary evidence for the acceptability and feasibility of a CBT-based intervention for DG and PG in adolescents. Preliminary data suggest that the treatment may be effective for DG but not PG. The participants showed less symptoms related to DG at the end of the treatment, and significantly, few participants met the criteria for game addiction according to the core approach.

## Data Availability

The data that support the findings of this study are available from the corresponding author upon reasonable request.
